# Influence of Titanium Content on the Microstructure and Tensile Behavior of Cold-Spray Additively Manufactured Copper-Titanium Composites

**DOI:** 10.3390/ma18225100

**Published:** 2025-11-10

**Authors:** Jia Cheng, Jibo Huang, Haifan Li, Kejie Zhang, Tao Chen, Haiming Lan, Renzhong Huang

**Affiliations:** 1 China Yangtze Power Co., Ltd., Yichang 443000, China; cheng_jia1@ctg.com.cn (J.C.); li_haifan@ctg.com.cn (H.L.); chen_tao3@ctg.com.cn (T.C.); 2 Guangzhou Institute of Hubei Chaozhuo Aviation Technology Co., Ltd., Guangzhou 510530, China; zhangkejie@cz-tec.com (K.Z.); lanhaiming@cz-tec.com (H.L.)

**Keywords:** cold-spray additive manufacturing, copper–titanium composite, titanium content, heat treatment, tensile properties, microstructure

## Abstract

Cold-spray additive manufacturing (CSAM) is an emerging solid-state deposition technology that effectively mitigates common defects associated with conventional thermal processes, such as oxidation, phase transformation, and residual stresses. In this study, copper–titanium (Cu-Ti) composite coatings were fabricated via high-pressure CSAM using mixed powders with Ti contents of 3, 6, and 10 wt.%. The influence of Ti content and post-heat treatment (350–400 °C) on the tensile properties of the composites was systematically investigated. The results indicate that the ultimate tensile strength (UTS) remained consistently within the range of 265–285 MPa under all conditions, showing only a mild positive correlation with Ti content. In contrast, ductility was significantly influenced by Ti addition, with elongation decreasing markedly as the Ti content increased. Notably, the composite with 3 wt.% Ti heat-treated at 400 °C exhibited a well-balanced combination of tensile strength (270 MPa) and ductility (20% elongation). These findings demonstrate that CSAM-fabricated Cu-Ti composites possess attractive mechanical properties, which can be tailored through Ti content and heat treatment.

## 1. Introduction

Copper is an indispensable metallic material in modern industry due to its exceptional electrical and thermal conductivity, mechanical workability, and balanced mechanical properties. It has found extensive applications in integrated circuits, power transmission, aerospace, and electrical contact components. With the rapid development of electronic packaging and nuclear energy sectors, there is an escalating demand for copper-based materials with superior comprehensive performance, including enhanced mechanical properties, wear resistance, arc erosion resistance, and tailored thermal expansion coefficients [1,2,3,4,5,6].

Conventional copper alloys struggle to meet these stringent requirements, whereas copper matrix composites (CMCs) offer viable solutions [7,8,9]. For instance, Cu-W composites exhibit high thermal conductivity (200 W·m^−1^·K^−1^) and low thermal expansion (6.5 × 10^−6^ K^−1^), making them ideal for heat dissipation in electronic packaging [10]; Cu-Al_2_O_3_ demonstrates high strength, wear resistance, and thermal stability while retaining conductivity [11,12]; Cu-ZnO coatings provide microbial inhibition for marine antifouling [13]; and Cu–diamond composites combine extreme thermal management capabilities with wear resistance [14,15].

Traditional coating techniques (e.g., thermal spraying, laser cladding) for fabricating CMCs face inherent limitations. Plasma spraying and HVOF often introduce porosity, oxide inclusions, and elemental segregation, degrading functional properties [16,17,18]. Laser cladding suffers from incomplete melting due to copper’s high reflectivity and thermal conductivity, resulting in poor interfacial bonding and high residual stresses [19,20]. These deficiencies necessitate alternative deposition strategies.

Cold-spray additive manufacturing (CSAM), a solid-state deposition technology, emerges as a promising solution. In CSAM, metallic powders (10–70 µm) are accelerated by preheated pressurized gas (300–1200 m/s) through a de Laval nozzle, impacting the substrate in a fully solid state. Particle bonding is achieved via severe plastic deformation rather than melting, thereby eliminating solidification-related defects such as oxidation, phase transformations, and high thermal stresses. This enables the production of dense, low-oxide coatings with near-net-shape dimensional accuracy, though the as-sprayed surface typically exhibits a roughness (Ra) in the range of 10–30 µm and may require post-processing for applications demanding a smooth finish. These advantages position CSAM as a uniquely suitable technique for copper-based composites [4,21,22,23,24].

Current research on CSAM-fabricated CMCs primarily focuses on systems such as Cu–Cr [25], Cu–Ni [26], Cu–Al [27], and Cu–W [28]. Notably, while Cu-Ti composites are attractive, their fabrication via CSAM remains largely unexplored, creating a significant knowledge gap. Specifically, the fundamental relationship between Ti content, the resulting microstructure, and the tensile mechanical properties in the CSAM system is unknown. Our prior work [29] demonstrated that optimizing propellant gas temperature (800 °C) and post-heat treatment (400 °C) synergistically enhanced the tensile properties of CSAM Cu-6 wt.% Ti composites, achieving a strength of 270 MPa and elongation of 15%—outperforming wrought pure copper (194 MPa) and annealed cold-sprayed Cu (170–190 MPa). Despite this progress, a systematic understanding of how titanium particle content regulates the microstructure and mechanical properties is still lacking.

While the introduction of Ti is expected to reduce electrical conductivity, this study targets applications where a combination of strength, ductility, and wear resistance is prioritized over maximum conductivity. The primary objective of this work is to establish a fundamental process–structure–property relationship for the CSAM Cu-Ti system, with a specific focus on the role of Ti content in governing the microstructural evolution and tensile behavior. A thorough understanding of this mechanical foundation is essential before exploring functional properties in future work.

Herein, we systematically investigate the effect of Ti particle content (3–10 wt.%) on the hardness and tensile behavior of CSAM Cu-Ti composites post-treated at 350–400 °C. By correlating microstructural evolution with mechanical responses, this work establishes a process–property relationship for Ti-reinforced copper composites, providing critical insights for deploying CSAM in high-performance component fabrication.

## 2. Materials and Methods

### 2.1. Material Preparation and Composite Design

**Figure 1:**
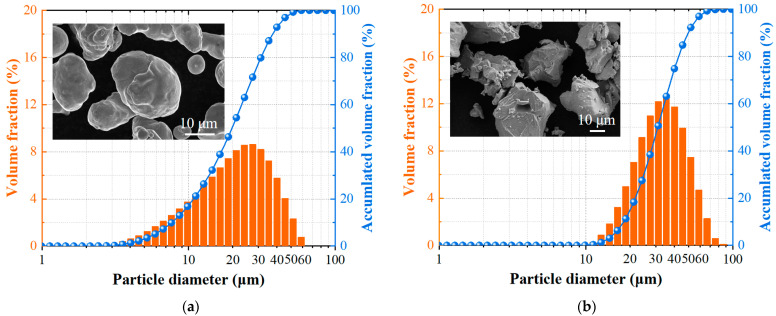
Particle size distribution and morphological characteristics of feedstock powders for copper–titanium composites: (**a**) copper powder, showing a near-spherical morphology; (**b**) titanium powder, exhibiting an irregular, angular morphology.

Commercial gas-atomized pure copper powder and plasma-rotary-atomized titanium powder were utilized. As detailed in Figure 1, the Cu powder exhibited a near-spherical morphology with a particle size distribution of 3–70 µm (D_50_ = 22.5 μm), while the Ti powder was irregularly shaped with sizes ranging from 10 to 80 µm (D_50_ = 35.1 μm). Three composite feedstocks with Ti contents of 3, 6, and 10 wt.% were blended (compared to 6 wt.% in prior work [29]) via mechanical mixing using a tumbling mixer (DRY-PL, Hefei Kejing Materials Technology Co., Ltd., Hefei, China) to ensure homogeneity.

### 2.2. Cold-Spray Deposition

Deposition was performed using a high-pressure cold-spray system (PCS-800, Plasma Giken Co., Ltd., Yorii, Japan) with high-purity (99.999%, 5 N) nitrogen as the propellant gas at 5 MPa. Based on previous optimization [29], the gas temperature was fixed at 800 °C to maximize particle deformation. Other parameters remained consistent: spray distance = 30 mm, nozzle traverse speed = 400 mm/s, and layer pitch = 2 mm. Cubic deposits (150 mm × 100 mm × 5 mm) were fabricated on grit-blasted 304 stainless steel substrates. Prior to deposition, the substrates were mechanically ground and polished to ensure a clean, oxide-free surface for initial particle adhesion. After the cold-spray deposition process, the composite coatings were separated from the substrates using wire electrical discharge machining (wire-EDM).

### 2.3. Heat Treatment

Specimens were heat-treated at 350 °C, 380 °C, and 400 °C for 2 h in a furnace (SXL-1400C, Shanghai Jujing Precision Instrument Manufacturing Co., Ltd., Shanghai, China), followed by furnace cooling.

### 2.4. Characterization Techniques

Microstructural characterization was conducted using a Rigaku SmartLab SE X-ray diffractometer (Rigaku, Tokyo, Japan) with Cu–Kα radiation operated at 40 kV and 40 mA. Scans covered a 2θ range of 20–80° at a rate of 2°/min. Scanning electron microscopy with energy-dispersive spectroscopy (SEM/EDS) analysis was performed on a Gemini SEM 300 instrument (Carl Zeiss AG, Cambridge, UK) using a 10 kV accelerating voltage. Prior to imaging, samples were metallographically etched for 15 s in a solution containing 5 g FeCl_3_, 10 mL HCl, and 100 mL H_2_O. Titanium particle distribution was quantified through image analysis of backscattered electron (BSE) SEM micrographs using ImageJ software (v1.53t, National Institutes of Health, Bethesda, MD, USA).

**Figure 2:**
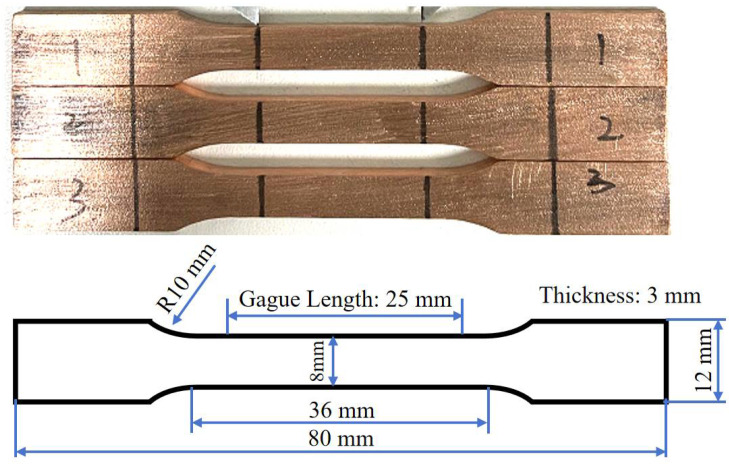
Geometry and dimensions of the tensile specimen, extracted perpendicular to the deposition direction from cold-sprayed Cu-Ti composites. The numbers 1, 2, and 3 marked on the specimen denote the three parallel samples.

Tensile properties were determined using ASTM E8 [30] sub-sized specimens machined perpendicular to the deposition direction via wire electrical discharge machining, as illustrated in Figure 2. Quasi-static tensile tests were carried out at a constant strain rate of 1 mm/min using an INSTRON 5982 universal testing machine (INSTRON, Norwood, MA, USA).

## 3. Results and Discussion

### 3.1. Microstructure

**Figure 3:**
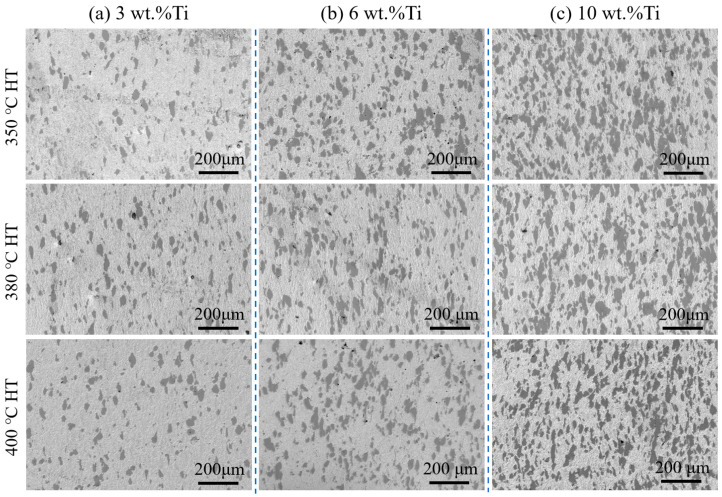
Microstructure of cold-sprayed Cu-Ti composites with varying Ti contents after heat treatment at 350 °C, 380 °C, and 400 °C: (**a**) 3 wt.%, (**b**) 6 wt.%, (**c**) 10 wt.%.

Figure 3 presents cross-sectional microstructures of cold-sprayed Cu-Ti composites with different Ti particle contents: 3 wt.% (a), 6 wt.% (b), and 10 wt.% (c). All specimens exhibit a fully dense structure without discernible pores or microcracks, a characteristic advantage of the cold-spray process attributable to intense plastic deformation upon impact [31]. The Ti particles (dark contrast) are uniformly dispersed in the copper matrix, retaining the irregular morphology of the original powder, and show excellent interfacial bonding with the copper phase. SEM observations reveal sharp and distinct interfaces between the Ti particles and the Cu matrix in the heat-treated composites, suggesting a primarily mechanical combination between the two phases without substantial interdiffusion, which is consistent with previous reports on cold-sprayed Cu-Cr composites [25]. The microstructures show no significant variation across the range of heat treatment temperatures (350–400 °C). As the Ti content in the feedstock powder increases, the corresponding volume fraction of Ti particles in the deposits also rises considerably.

**Figure 4:**
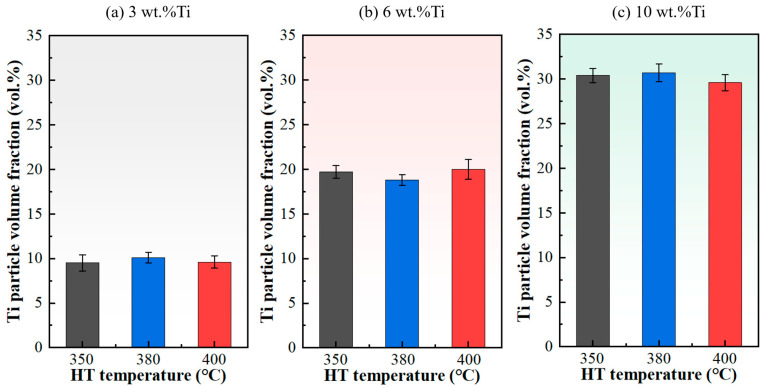
Volume fraction of Ti particles in cold-sprayed Cu-Ti composites as a function of heat treatment for different Ti contents: (**a**) 3 wt.%, (**b**) 6 wt.%, (**c**) 10 wt.%.

The volume fractions of Ti particles in the composites, corresponding to the different initial Ti contents in the powders, were quantitatively analyzed, and the results are summarized in Figure 4. The measured Ti volume fraction is approximately 10% in the sample prepared with 3 wt.% Ti powder, 20% for the 6 wt.% Ti condition, and reaches about 30% for the 10 wt.% Ti sample. The heat treatment temperature, within the range of 350–400 °C, exhibits no notable effect on the retained Ti content within the coatings.

**Figure 5:**
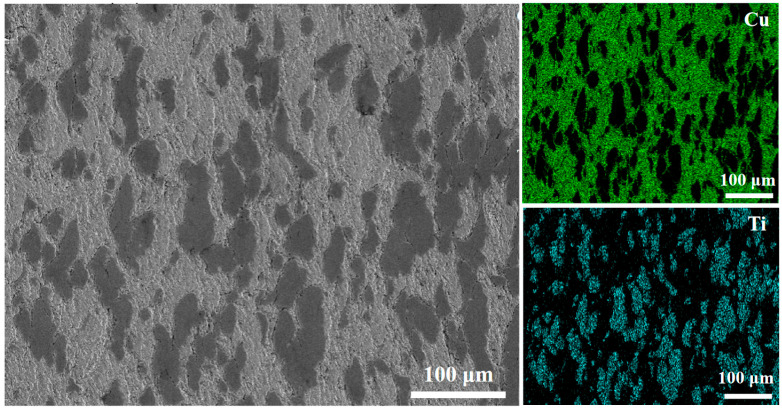
EDS elemental mapping of the cold-sprayed Cu–Ti composite (10 wt.% Ti) after heat treatment at 400 °C, showing the distribution of Ti particles.

To further clarify the distribution of Ti within the composite, EDS elemental mapping was conducted on a specimen containing 10 wt.% Ti after heat treatment at 400 °C; the results are presented in Figure 5. The SEM micrograph and corresponding elemental maps confirm that the light regions correspond to copper, while the darker, particulate regions are titanium. The sharp interfaces between these regions provide further evidence that no significant diffusion occurred between Cu and Ti during either the cold-spray deposition or the subsequent heat treatment. The EDS analysis also confirms that the Ti volume fraction is approximately 30%, which is consistent with the results obtained from image analysis of the SEM micrographs.

**Figure 6:**
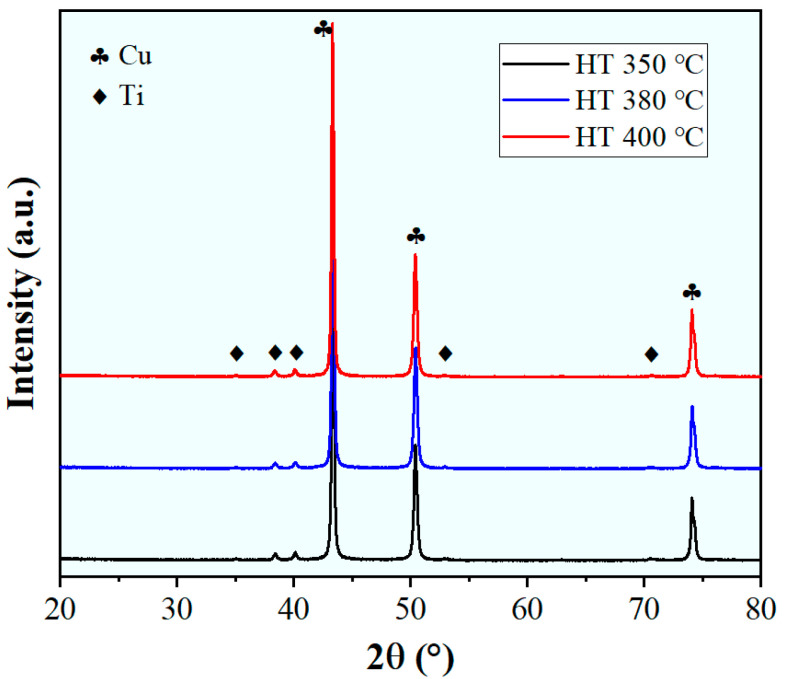
XRD patterns of the cold-sprayed Cu-10 wt.% Ti composite after heat treatment at 350 °C, 380 °C, and 400 °C.

The phase composition and stability of the cold-sprayed Cu-10 wt.% Ti composite under different heat treatment conditions were evaluated by XRD, as presented in Figure 6. The XRD patterns for all conditions exhibit exclusively the characteristic diffraction peaks of pure copper (Cu, FCC structure) and pure titanium (Ti, HCP structure). No additional peaks corresponding to intermetallic compounds (e.g., CuTi, CuTi_2_, Cu_3_Ti) or oxides/nitrides (e.g., TiO_2_, CuO, TiN) are detectable within the instrument’s resolution limit. This unequivocally demonstrates that no interfacial reaction or interdiffusion occurred between the Cu matrix and the Ti particles during the cold-spray process or the subsequent heat treatments up to 400 °C, which aligns with the phase stability observed in cold-sprayed Cu-Cr composite deposits by Wu et al. [25]. The absence of intermetallic compounds, as confirmed by XRD, aligns with the thermodynamic expectation that significant interdiffusion between Cu and Ti requires temperatures substantially higher than the 350–400 °C range used in this study. This confirmation of phase stability is crucial, as it ensures that the observed mechanical properties can be attributed solely to the mechanical interplay between the Cu matrix and the Ti particles, rather than to complications from interfacial reactions [32,33].

The microstructure analysis confirms the successful fabrication of a physically mixed, metallurgically stable Cu-Ti composite via CSAM. The absence of deleterious brittle intermetallic phases, combined with the thermally induced recovery of the Cu matrix, provides the microstructural foundation for the improved mechanical properties, particularly the ductility restoration after the 400 °C heat treatment.

### 3.2. Tensile Properties

**Figure 7:**
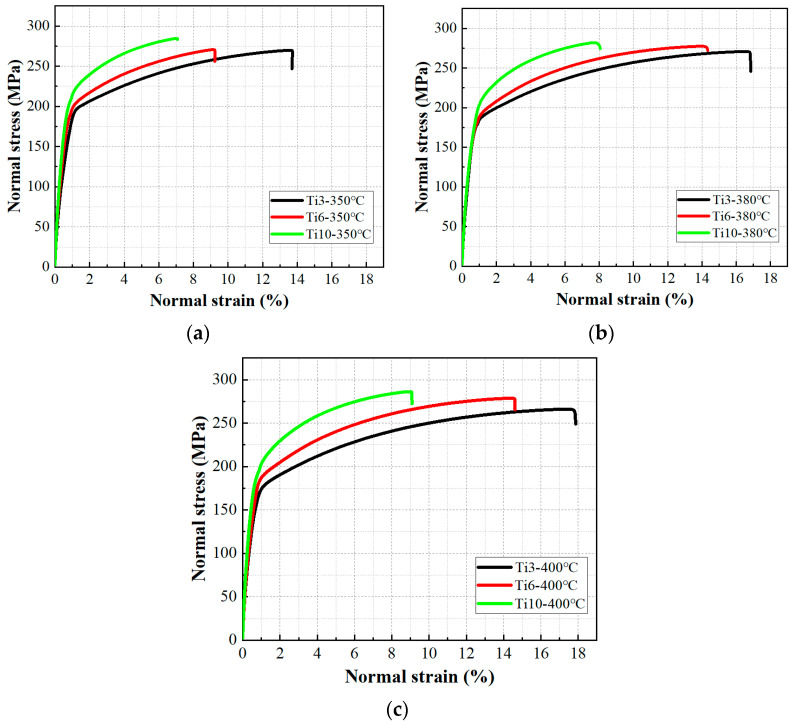
Engineering stress–strain curves of cold-sprayed Cu-Ti composites with different Ti contents (3, 6, and 10 wt.%) after heat treatment at (**a**) 350 °C, (**b**) 380 °C, and (**c**) 400 °C.

The tensile performance of the cold-sprayed Cu-Ti composites was systematically evaluated to understand the effects of titanium content and heat treatment temperature. Representative engineering stress-strain curves are presented in Figure 7, revealing consistent ductile fracture behavior across all samples. Each curve displays a linear elastic region followed by a plastic strain-hardening stage, indicative of dislocation-mediated deformation in the copper matrix. The absence of an abrupt fracture confirms the lack of brittle intermetallic phases, as supported by XRD analysis.

All composites exhibit conventional monotonic strain hardening without the sigmoidal curvature typically associated with twin-mediated hardening in some thermomechanically processed HCP alloys [34]. This confirms that deformation twinning is not an active mechanism in our FCC Cu matrix. The high density of Cu-Ti interfaces and the residual strain states from CSAM create a microstructure where dislocation glide and accumulation are dominant from the very onset of plasticity, overwhelming any potential for a transition into a deformation regime governed by twinning.

**Figure 8:**
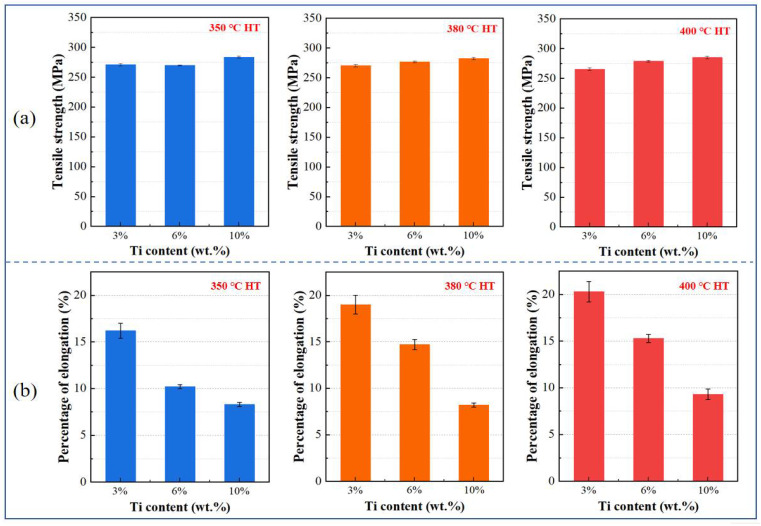
Tensile properties of cold-sprayed Cu-Ti composites as a function of Ti content: (**a**) tensile strength and (**b**) elongation after heat treatment at 350 °C, 380 °C, and 400 °C.

Quantitative analysis of the tensile properties is summarized in Figure 8, which clearly illustrates the relationships between Ti content, heat treatment temperature, and mechanical performance. As shown in Figure 8a, the ultimate tensile strength (UTS) values for all composites remained within a relatively narrow range of 250–300 MPa, demonstrating limited sensitivity to Ti content. It is noteworthy that the tensile strength of the cold-sprayed Cu-Ti composites in this study is significantly higher than that of cold-sprayed pure copper reported in numerous studies. For instance, Boyle et al. [35] reported that copper deposits prepared under similar cold-spray conditions and heat-treated between 350–600 °C exhibited a UTS of approximately 200 MPa, while Gärtner et al. [18] also found that the optimum UTS of nitrogen-sprayed copper deposits after various heat treatments was around 200 MPa. The Ti10 composite reached approximately 290 MPa at 400 °C, while the Ti3 composite achieved about 270 MPa under the same conditions. This modest strength increase with Ti content suggests that interfacial mechanical interlocking, rather than metallurgical bonding, dominates load transfer between the matrix and reinforcement.

In contrast to the strength behavior, elongation exhibited strong dependence on both parameters, as clearly demonstrated in Figure 8b. The Ti content has a strong and consistent impact on the ductility of the composites, and the difference in elongation between composites with different Ti contents becomes more obvious as the heat treatment temperature increases. At 350 °C, elongation decreases dramatically with increasing Ti content: from 16.0% for Ti3 to 10.0% for Ti6, and further to 8.3% for Ti10, corresponding to a 48.1% reduction from the lowest to the highest Ti content. This trend persists at elevated temperatures, with absolute values increasing due to thermal recovery effects but maintaining the same hierarchical order. At 400 °C, the Ti3 composite achieves exceptional ductility (20.3%), while Ti6 and Ti10 reach 15.3% and 9.3%, respectively—still reflecting a 54.2% reduction across the Ti content range.

The significant ductility reduction with increasing Ti content stems from micromechanical mechanisms directly tied to the microstructure. The mechanical data-specifically, the negligible strengthening coupled with a severe, temperature-independent embrittlement-lead to a critical conclusion: the Ti particles function primarily as non-deforming inclusions rather than as effective strengthening reinforcements. As the Ti content rises, these rigid particles impose a potent interfacial constraint on the plastic flow of the Cu matrix. This constraint shortens the mean free path for dislocation motion and promotes stress concentration, ultimately limiting the matrix’s ability to accommodate large-scale plastic deformation [8]. This mechanism, governed by particle volume fraction, overrides the benefits of matrix recovery from heat treatment. It is noteworthy that this phenomenon, while well-established for metal matrix composites in general, appears to be under-explored specifically in cold-sprayed HCP composites with brittle second phases. The behavior observed here in our FCC Cu-Ti system thus provides a clear reference point for understanding particle-induced embrittlement in solid-state additively manufactured composites.

Heat treatment temperature modulates ductility, but its efficacy is strongly dependent on Ti content. The Ti3 composite exhibits the most significant improvement, with elongation increasing from 16.0% after being heat treated at 350 °C to 20.3% after being heat treated at 400 °C (a 26.9% enhancement), driven by thorough residual stress relief and recovery/recrystallization in the Cu matrix [32,36]. The Ti6 composite also benefits, with elongation rising from 10.0% to 15.3% (a 53% increase) as thermal energy facilitates partial mitigation of deformation constraints from Ti particles (20 vol.%). In contrast, the Ti10 composite shows limited improvement (from 8.3% to 9.3%, a 12% increase) because its dense Ti particle network (30 vol.%) imposes severe restrictions on plastic flow, effectively overriding the benefits of matrix recovery.

The comprehensive data in Figure 8 enable direct comparison of strengthening and ductilizing effects across compositions and treatment conditions, demonstrating the tunability of mechanical properties through Ti content and heat treatment. Low-Ti composites (e.g., Ti3) are ideal for applications demanding high ductility (e.g., deformable thermal management components), while high-Ti composites (e.g., Ti10) offer enhanced strength with acceptable toughness—making them suitable for structural applications where moderate deformation resistance is required. These findings establish a framework for tailoring cold-sprayed Cu-Ti composites to specific engineering needs.

### 3.3. Fracture Behavior Analysis

#### 3.3.1. Fracture Characteristics

**Figure 9:**
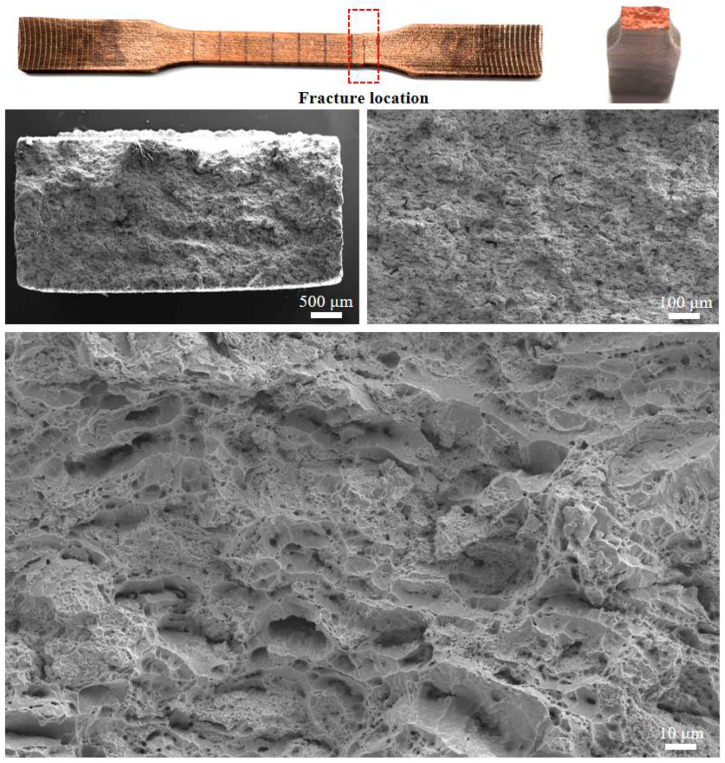
Representative macroscopic views of tensile-fractured cold-sprayed Cu-Ti composites and fracture morphology characteristics, with the 3 wt.% Ti specimen after 400 °C heat treatment as the illustrative example.

Figure 9 presents representative macroscopic and microscopic fracture features of the cold-sprayed Cu-Ti composites, illustrated here using the 3 wt.% Ti specimen after 400 °C heat treatment. Macroscopically, all tensile specimens fractured following uniform elongation without visible necking, yielding flat fracture surfaces with no evidence of localized deformation or defect-induced failure (e.g., tearing or bulging). This type of fracture surface is consistent with the typical macroscopic morphology reported for cold-sprayed copper deposits [36], and suggests a homogeneous microstructure with evenly distributed plastic deformation.

At higher magnification, the fracture surface exhibits a uniformly dimpled morphology, characteristic of microvoid coalescence and ductile fracture. The presence of abundant dimples confirms that failure occurred through extensive plastic deformation in the copper matrix, consistent with the measurable elongations reported in Section 3.2. Additionally, localized cleavage features are observed in certain regions, which may be attributed to the presence of hard Ti particles that act as stress concentrators, facilitating brittle fracture in these limited areas. This mixed-mode fracture behavior reflects the influence of titanium reinforcement on the overall ductility of the composite.

**Figure 10:**
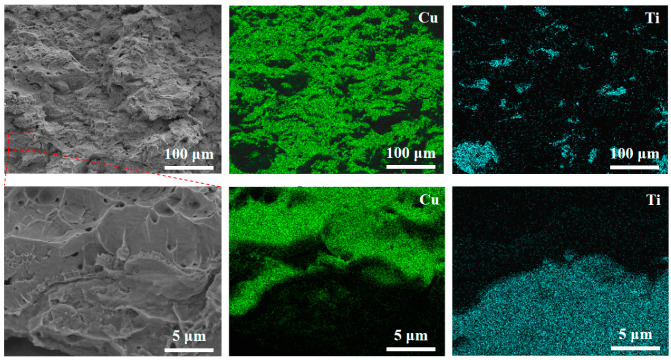
EDS elemental mapping of the fracture surface of the cold-sprayed Cu–Ti composite, revealing the distribution of Cu and Ti.

Figure 10 shows EDS elemental mapping of the fracture surface, confirming the distribution of Ti particles. No distinct morphological differences are observed between Ti-rich and Cu-rich regions, suggesting coordinated deformation between the two phases. High-magnification imaging reveals intact Cu-Ti interfaces with no debonding or microcracks, indicating good interfacial bonding achieved via cold spraying.

Furthermore, high-magnification examinations of the regions adjacent to the fracture path did not reveal clear microstructural evidence of new phase formation. While this observation does not preclude the possibility of subtle interfacial changes, it supports the interpretation that the embrittling effect of Ti addition is primarily a consequence of mechanical constraint.

#### 3.3.2. Effect of Ti Content on Fracture Behavior

**Figure 11:**
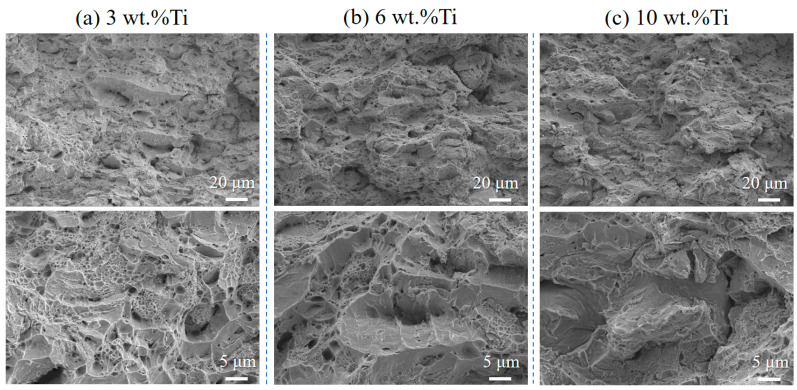
Fracture surface morphology of cold-sprayed Cu-Ti composites with varying Ti contents after tensile testing and heat treatment at 400 °C: (**a**) 3 wt.%, (**b**) 6 wt.%, and (**c**) 10 wt.%.

Figure 11 compares the fracture surfaces of composites with 3, 6, and 10 wt.% Ti after 400 °C heat treatment. Although all samples exhibit dimpled fracture, the size and density of dimples decrease with increasing Ti content. This trend is consistent with the reduction in elongation presented in Figure 8b, where higher Ti contents led to lower ductility.

The evolution of dimple morphology with Ti content provides a direct qualitative link to the energy dissipation capacity of the composites. The larger, deeper dimples in low-Ti composites (Figure 11a) are characteristic of extensive plastic deformation and high energy absorption prior to fracture, which correlates with their superior elongation. Conversely, the shallower, finer dimples in high-Ti composites (Figure 11c) indicate constrained plasticity and reduced energy absorption. This trend aligns with an energy-based framework for fracture analysis, wherein the total strain energy density is intrinsically linked to the topography of the fracture surface [34]. The quantitative relationship between parameters such as areal roughness, void volume, and energy density, as demonstrated by Macek et al. for additively manufactured steel [34], provides a compelling methodology for future work to build upon the qualitative correlations established here.

**Figure 12:**
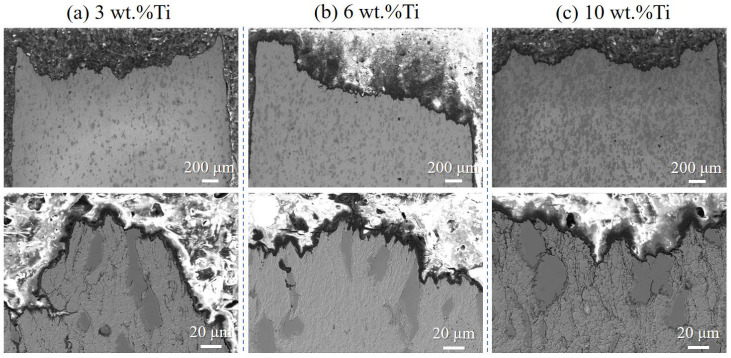
Fracture surface cross-sections of cold-sprayed Cu-Ti composites with varying Ti contents after tensile testing and heat treatment at 400 °C: (**a**) 3 wt.%, (**b**) 6 wt.%, and (**c**) 10 wt.%.

Cross-sectional views of the fracture path (Figure 12) confirm that cracking occurs primarily within the copper matrix, with no interfacial failure or Ti particle fracture. The increasing volume fraction of Ti particles (from ~10 vol.% to ~30 vol.%) restricts plastic flow in the Cu matrix, shortening the mean free path for dislocation motion and inhibiting void growth and coalescence. This microstructural constraint mechanism aligns well with the macroscopic tensile results, supporting the conclusion that Ti content modulates—but does not fundamentally alter—the ductile fracture mode.

#### 3.3.3. Effect of Heat Treatment Temperature on Fracture Behavior

**Figure 13:**
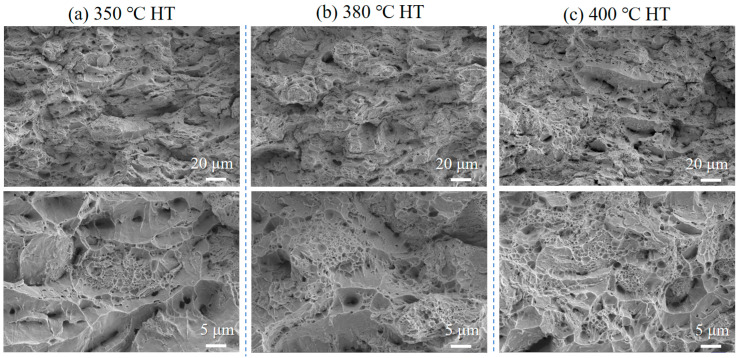
Fracture surface morphology of the cold-sprayed Cu-3 wt.% Ti composite after tensile testing following heat treatment at (**a**) 350 °C, (**b**) 380 °C, and (**c**) 400 °C.

Figure 13 shows fracture surfaces of the Cu-3 wt.% Ti composite after heat treatment at 350 °C, 380 °C, and 400 °C. With increasing temperature, the dimples become larger and more numerous, reflecting enhanced plastic deformation capacity. This correlates with the improved elongation values reported in Section 3.2, where higher heat treatment temperatures led to greater ductility recovery.

**Figure 14:**
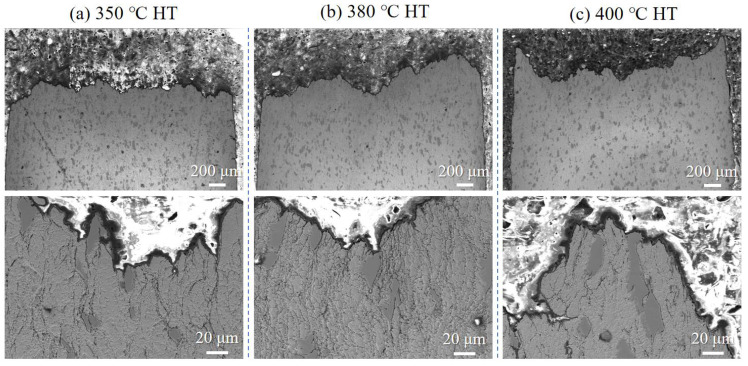
Cross-sectional microstructure of the fracture surface of the cold-sprayed Cu-3 wt.% Ti composite after tensile testing under different heat treatment temperatures: (**a**) 350 °C, (**b**) 380 °C, and (**c**) 400 °C.

Cross-sectional analysis (Figure 14) reveals that the fracture remains confined to the Cu matrix across all temperatures, with no degradation in Cu-Ti interfacial bonding. The improvement in ductility is attributed to the recovery of the copper matrix and relief of residual stresses, which facilitate more uniform plastic flow and void formation. These observations confirm that heat treatment enhances ductility without compromising interfacial integrity or fracture mode.

## 4. Conclusions

This study systematically investigated the effect of titanium particle content (3, 6, and 10 wt.%) and heat treatment temperature (350, 380, and 400 °C) on the microstructure and tensile properties of cold-spray additively manufactured Cu-Ti composites. The main findings are summarized as follows:

(1) Dense and defect-free Cu-Ti composites were successfully fabricated by cold spraying, with Ti particles uniformly dispersed in the copper matrix. The absence of intermetallic compounds and oxides confirms that the CSAM process facilitates solid-state deposition without undesirable phase reactions. Fractographic examination revealed a ductile fracture mode across all composites, characterized by dimpled surfaces and absence of interfacial debonding, demonstrating strong mechanical stability and interfacial integrity of the Cu–Ti interfaces.
(2) The ultimate tensile strength (UTS) showed limited sensitivity to Ti content, remaining in the range of 265–285 MPa across all conditions. This suggests that strength is dominated by the Cu matrix and interfacial mechanical bonding rather than Ti reinforcement. Ductility was significantly influenced by Ti content, with elongation decreasing from 20.3% (3 wt.% Ti) to 9.3% (10 wt.% Ti) after 400 °C treatment. This reduction is attributed to the constraint effect of Ti particles on dislocation motion and plastic flow in the Cu matrix.
(3) Heat treatment within the range of 350–400 °C not only enhanced ductility through recovery and stress relief in the copper matrix—particularly in low-Ti composites—but also contributed to a moderate improvement in strength. The ultimate tensile strength exhibited a slight but consistent increase with higher treatment temperatures across all Ti contents. The optimum combination of strength and ductility was achieved in the 3 wt.% Ti composite after 400 °C treatment.

The demonstrated combination of strength and ductility suggests potential for these composites in applications such as specialized structural parts. To fully assess their potential in specific applications, future work should encompass the characterization of functional properties (e.g., electrical/thermal conductivity) and mechanical performance under different loading conditions, such as bending, dynamic, or cyclic loading.

## Data Availability

The original contributions presented in this study are included in the article. Further inquiries can be directed to the corresponding authors.
